# Multi- and Transgenerational Effects of Silver Ions (Ag^+^) in the ng/L Range on Life Cycle Parameters and Population Growth of the Midge *Chironomus riparius* (Diptera, Chironomidae)

**DOI:** 10.3390/toxics13100855

**Published:** 2025-10-10

**Authors:** Jingyun Ding, Stefanie Krais, Zequn Li, Rita Triebskorn, Heinz-R. Köhler

**Affiliations:** 1 Animal Physiological Ecology, University of Tübingen, Auf der Morgenstelle 5, D-72076 Tübingen, Germany; stefanie.krais@uni-tuebingen.de (S.K.); zequn.li@student.uni-tuebingen.de (Z.L.); rita.triebskorn@uni-tuebingen.de (R.T.); heinz-r.koehler@uni-tuebingen.de (H.-R.K.); 2 Steinbeis Transfer Center for Ecotoxicology and Ecophysiology, Blumenstrasse 13, D-72108 Rottenburg, Germany

**Keywords:** silver, silver ion, Ag^+^, *Chironomus riparius*, multigenerational toxicity, transgenerational effects, population growth, aquatic ecotoxicology

## Abstract

Silver (Ag) is widely released into aquatic environments through industrial and municipal discharges, with concentrations often reaching toxic levels for aquatic organisms. Its further extensive use in antimicrobials, especially during the COVID-19 pandemic, has increased environmental inputs. As Ag^+^ is the most toxic form of Ag, understanding its ecological risks remains critical for environmental regulation and ecosystem protection. Thus, we investigated multigenerational and transgenerational toxicity of Ag^+^ as AgNO_3_ on the ecologically important species midge *Chironomus riparius* using two complementary long-term life-cycle experiments. Experiment 1 simulated exposures with pulsed high environmentally relevant concentrations and recovery phases (nominal 3 µg/L), while Experiment 2 assessed continuous low environmentally relevant concentrations (nominal 0.01, 0.1, 1 and 3 µg/L) across four exposed generations of *C. riparius* followed by three recovery generations. Endpoints included survival, development, reproduction, growth as well as the population growth rate (PGR). Continuous Ag^+^ exposure produced cumulative increases in mortality and declines in emergence, reduced fertility and eggs per rope, delayed development (especially in females), and progressive reductions in PGR. Notably, adverse effects emerged or intensified over generations and were detectable at very low concentrations: some reproductive and survival endpoints showed significant impairment at the European Union’s environmental quality standard (EU-EQS) level (0.01 µg/L) by the fourth generation, while transgenerational effects persisted at ≥0.1 µg/L. Partial recovery occurred after removal of contamination at the lowest concentrations but not after higher exposures. The present study not only indicates that chronic, low-level Ag^+^ contamination can produce persistent, population-level adverse impacts on *C. riparius*, but also underscores the necessity for long-term ecological assessments to establish more protective standards and maintain ecosystem stability.

## 1. Introduction

Silver (Ag) is widely utilized in diverse applications, including electronics, batteries, catalysts, and jewelry [1]. Aquatic environments often receive Ag primarily from municipal and industrial wastewater treatment plant effluents (e.g., from the photographic industry), establishing its potential as a main indicator of urban pollution [2]. Reported Ag concentrations in surface waters range from pg/L to μg/L [3,4], levels that can be toxic to aquatic life. Maximum observed concentrations reach about 6.0 µg/L in groundwater, 260 µg/L in rivers, and 300 µg/L in treated photoprocessing wastewater [5]. Ag is regulated under the European Commission Biocidal Products Directive and classified as a priority contaminant in natural waters by the US Environmental Protection Agency (U.S. EPA) [6,7]. Recently, the broad-spectrum antimicrobial activity of silver ions (Ag^+^) and silver nanoparticles (AgNPs) [8,9] has led to their extensive use as antimicrobial products. A survey of 55 biocide companies found approximately 91% of Ag is used as Ag^+^, with only about 9% as AgNPs [10]. Importantly, as with other metals, the ionic form Ag^+^ is of greatest concern for bioavailability and toxicity, serving as the principal driver of silver’s environmental effects [4].

Particularly during the long-term worldwide COVID-19 pandemic in the recent years, AgNP-incorporated polycotton fabrics proved effective against SARS-CoV-2 [11]. Ag-coated masks were also shown to inhibit the virus [12], and Ag-based sanitizers and disinfectants were widely used for hand hygiene and inanimate surfaces disinfection [13]. This extensive use of silver as a disinfectant led to increasing releases of free Ag^+^ ions from AgNPs into the environment [14,15]. Consequently, numerous studies investigated toxicity of AgNPs [16,17] and confirmed that Ag^+^ is the most toxic form of Ag for aquatic organisms [18].

In general, Ag occurs naturally in the environment at low concentrations, as dissolved salts like silver nitrate (AgNO_3_) [3,18,19]. Dissolved Ag^+^ concentrations typically remain below 0.2 μg/L, although this is highly dependent on environmental conditions [20], with elevated levels often observed near industrial discharge zones [21]. As part of the substance evaluation for Ag under the European Union Registration, Evaluation, Authorisation, and Restriction of Chemicals (REACH) regulation (EC) No. 1272/2008 [22]. Mertens et al. (2019) provided further justification for read-across from soluble Ag salts to AgNPs [1]. Regulatory frameworks for Ag pollution are predominantly based on research data on the ionic Ag^+^ form [23,24,25,26]. A regulatory standard (AA-QS_eco, fw_) for Ag and its compounds set to 0.01 µg/L has been endorsed by Scientific Committee on Health, Environmental and Emerging Risks (SCHEER) [27]. Elevated metal pollution correlates directly with declines in benthic communities [28,29,30]. Additionally, increased littering of personal protective equipment (PPE) during the COVID-19 pandemic [31] has emerged as a significant source of Ag^+^ contamination, threatening the ecological balance of aquatic ecosystems.

In freshwater ecosystems, insects exhibit the highest species diversity and play crucial functional roles. Diptera, Coleoptera, and Trichoptera are the dominant orders [32]. Chironomidae, the most diverse family within the order Diptera, dominate benthic communities in abundance. These ubiquitous insects represent a key trophic resource for both vertebrate and invertebrate species [33,34,35]. *Chironomus riparius*, a widespread species in Europe, North America and Asia, is an ecologically important taxon within the Chironomidae family and is a key component of both benthic and terrestrial food webs [36,37]. Furthermore, *C. riparius* is a well-established model organism extensively employed in ecotoxicological studies and is approved for testing by both the U.S. EPA and OECD [38,39]. Its ecological relevance, short life cycle, ease of laboratory culture, and high sensitivity to contaminants make it particularly suitable for risk assessment, and it is routinely recommended in standardized sediment and water toxicity tests [39]. It is also recognized as a relevant bioindicator species for metal accumulation [40,41,42]. Previous research on Ag toxicity to *C. riparius* indicates that Ag^+^ is the primary toxicant released from AgNPs [43]. Classical life-history endpoints (survival, reproduction, growth, development) remain the most reliable and commonly used measures for evaluating population-relevant toxic responses [36]. Considering the worldwide ecological importance of *C. riparius* and its standard use in ecotoxicology, assessing its risks under Ag^+^ exposure is therefore both necessary and urgent.

Up to now, most ecotoxicological studies only consider short-term effects and multigenerational assessments are not commonly conducted. [44,45,46]. Traditional standard ecotoxicological test guidelines predominantly rely on acute and single-generation laboratory bioassays [1,47]. Consequently, conventional approaches may fail to predict effects in chronically contaminated ecosystems, as short-term tests can overlook persistent or progressively worsening multigenerational impacts within natural populations [47]. Moreover, the COVID-19 pandemic led to the extensive use and disposal of Ag-containing medical and protective products, substantially increasing Ag^+^ contamination in aquatic environments. As a result, *C. riparius* populations worldwide have likely experienced continuous Ag^+^ exposure over an extended period. This underscores the importance of implementing long-term, full life-cycle tests for *C. riparius*, which enable the evaluation of toxicity during sensitive developmental stages.

In natural ecosystems, organisms are typically exposed to persistent chemicals at sublethal concentrations over multiple generations. Contaminant exposure can occur via episodic pulses or discharges of relatively high, transient concentrations [48], or through persistent low-level background contamination [49]. However, previous long-term studies have predominantly focused on continuous multigenerational exposure to toxicants [50,51,52,53], often neglecting pulsed exposure scenarios [48].

Therefore, we developed two complementary multigenerational and transgenerational experimental series with *C. riparius* to realistically examine both continuous and pulsed exposure scenarios at environmentally relevant sublethal Ag^+^ concentrations. Our aim was to provide insight into the real-world threats of chronic Ag^+^ exposure to *C. riparius* and to detect effects potentially overlooked by standard acute or single-generation tests. Ultimately, these experiments contribute to evaluating ecologically relevant population-level consequences and reducing uncertainty in the ecological risk assessment for this taxon.

## 2. Materials and Methods

### 2.1. The Model Organism and Culture Conditions

The *C. riparius* population used in this study was established over a long period of cultivation in the laboratory of the Animal Physiological Ecology research group at the University of Tuebingen (Germany). The stock of *C*. *riparius* originated from three different institutions with independent long-term cultures (LimCo International, Constance, Germany; University of Joensuu, Finland; Universidade de Coimbra, Portugal). This approach could prevent genetic impoverishment and is beneficial for long-term toxicity studies related to the ecological aspects [50]. We maintained these lines under standard conditions and without introgression from local wild populations. Specifically, the stock culture was maintained in plastic basins, containing a bottom layer of fine quartz sand, covered by active carbon-filtered and dechlorinated tap water with continuous, gentle aeration. Larvae were fed fine fish food TetraMin^®^ (Tetra, Melle, Germany) every other day, and half of the water volume was changed once per week. All basins were covered by breeding cages to allow adult midges to swarm and mate. A total of five breeding cages were kept in a climate chamber at 21 ± 0.5 °C with a 16:8 h light/dark photoperiod.

### 2.2. Test Chemical

Silver nitrate (AgNO_3_) powder used in this study was obtained from Sigma-Aldrich (Merck, Darmstadt, Germany, purity ≥ 99.0%). A stock solution of 500 mg AgNO_3_ in 1 L of deionized water was prepared. The AgNO_3_ stock solution was stored in brown bottles wrapped in aluminum foil to protect them from light. Exposure concentrations were obtained by diluting required amounts of the stock dispersion in ISO medium (control condition) [54]. Specifically, four stock solutions were prepared in deionized water: CaCl_2_·2H_2_O (11.76 g/L), MgSO_4_·7H_2_O (4.93 g/L), NaHCO_3_ (2.59 g/L), and KCl (0.23 g/L). Then, for each liter of ISO medium, 25 mL of each stock was added to 900 mL of deionized water, and the mixture was brought to a final volume of 1 L. The prepared ISO medium was maintained at the experimental temperature (21 ± 0.5 °C) prior to use. AgNO_3_ test solutions were also freshly prepared at the same temperature as the experiments. Prior to the experiments, test vessels were filled with quartz sand (sediment) and saturated with the corresponding new test solutions for 24 h to saturate potential adsorption sites [55]. Subsequently, the solutions were carefully removed, leaving the sediment in the test vessels. Then all vessels were refilled with ISO medium (control) or the corresponding AgNO_3_ test solutions to start the experiments.

### 2.3. Experiment 1: Simulation of Contamination Scenarios with Multiple Generations Exposed to Pulses of Contamination and to Recovery Phases

**Figure 1:**
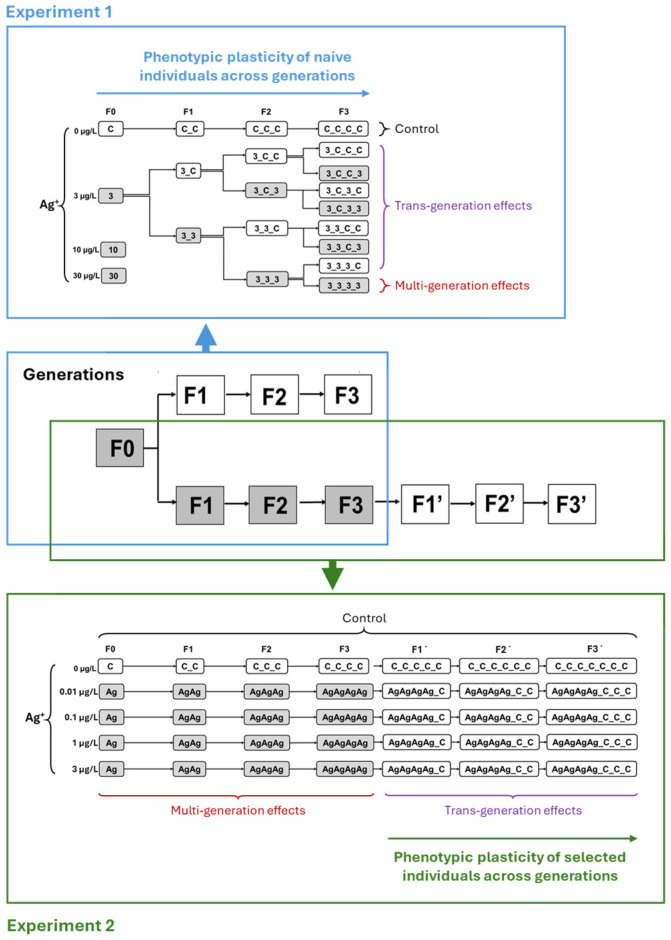
Overall long-term experimental design, combining aspects of multigenerational and transgenerational toxicity of Ag^+^ to *Chironomus riparius*. The zones marked with blue frames refer to the detailed experimental design regime for a total of four generations (F0–F3) in Experiment 1 (simulation of contamination scenarios with multiple generations exposed to pulses of contamination and recovery phase). The zones marked with green frames refer to the detailed experimental design regime for a total of seven generations (F0–F3 and F1′–F3′) in Experiment 2 (simulation of contamination scenarios with multiple generations of continuous exposure to low concentrations of Ag^+^ and subsequent recovery).

Experiment 1 was conducted at relatively high Ag^+^ concentrations to evaluate its potential to induce multigenerational effects. First of all, to determine an appropriate Ag^+^ concentration for long-term multigenerational toxicity experiment, Prior to this, a 144 h range-finding test (nominal 0, 0.5, 5, 25, 50, 75, 100, 150, 300 µg/L Ag^+^) was performed according to OECD guidelines (extended from the standard 48 h to 144 h in our study) [56,57] to identify an appropriate concentration for the further long-term study. To ensure that all larvae used in this pre-experiment were at the same age at the start of the experiment, fresh egg ropes (<24 h) were collected from the stock breeding cages and reared in a separate container for 23 days prior to the beginning of the pre-experiment [55]. Age stages were visually determined according to Day et al. [58] using a stereomicroscope, and only instars L3 and L4 were used [55]. Based on the mortality data obtained from this 144 h pre-experiment, the 10% lethal concentration (LC_10_, 144 h) of Ag^+^ (28.846 µg/L) was referred to establishing four nominal Ag^+^ concentration groups (0, 3, 10, and 30 µg/L) for the long-term Ag^+^ exposure study. They were named “C”, “3”, “10”, and “30”, respectively (Figure 1). The long-term life-cycle test was carried out basically following the OECD guideline [39]. Each treatment group consisted of five replicate glass containers (600 mL), maintained under the same conditions as the stock *C. riparius*. Three days before beginning with the parental generation (F0), 20 glass containers (5 per treatment, including controls) were prepared, each with 80 g of fine quartz sand and 350 mL of test medium (0, 3, 10, or 30 µg/L Ag^+^). All cultures, including controls, were initiated simultaneously from a single batch of 20 egg ropes collected from the stock culture. Egg ropes were placed individually in 24-well plates; each well contained 2 mL of the corresponding ISO medium (control condition). The first-instar larvae from ten fully hatched egg ropes (<48 h) were used to initiate the experiment. For each generation, 25 first-instar larvae were introduced into each glass container. Aeration was stopped for 24 h to allow the larvae to burrow into the sediment, after which aeration was supplied. Containers were inspected daily for adult emergence, and emergence timing and sex were recorded throughout the 28-day generation period. The daily emerged adults from all replicates were collected daily using an aspirator and transferred into breeding cages (one per treatment). Within these cages, they were allowed to swarm and mate. Each breeding cage contained a 1 L container of ISO medium (control water) as an oviposition site, and freshly laid egg ropes were collected from these containers daily. Collected egg ropes of each Ag^+^ concentration treatment group were individually transferred into corresponding 24-well plates. Each well of 24-well plates of each Ag^+^ treatment contained 2 mL test medium accordingly. The plates were stored at 4 °C [59]. This method of temporary low-temperature storage for collected egg ropes followed by unified larval hatchery ensures maximum synchronization of the multigenerational experiments among all treatment groups. Therefore, five hatched egg ropes of each treatment were randomly selected from the plate of different treatment groups at the same day. Thus, for each treatment, 1 For each treatment, 125 first-instar larvae (from five hatched egg ropes) were randomly allocated to five freshly prepared test vessels (25 first-instar larvae per vessel). Test solutions, quartz sand, and vessels were renewed ed between generations but not during a generation. Evaporation of water in the test container was compensated for by adding deionized water to the original volume (350 mL) [39,50]. Every day, 0.25 mg of fine fish food TetraMin^®^ (Tetra, Germany) was supplied per larvae. The long-term experiment was conducted in a climate chamber with a temperature of 21.0 °C ± 0.5 °C with a photoperiod of 12:12 h light/dark [60].

**Figure 2:**
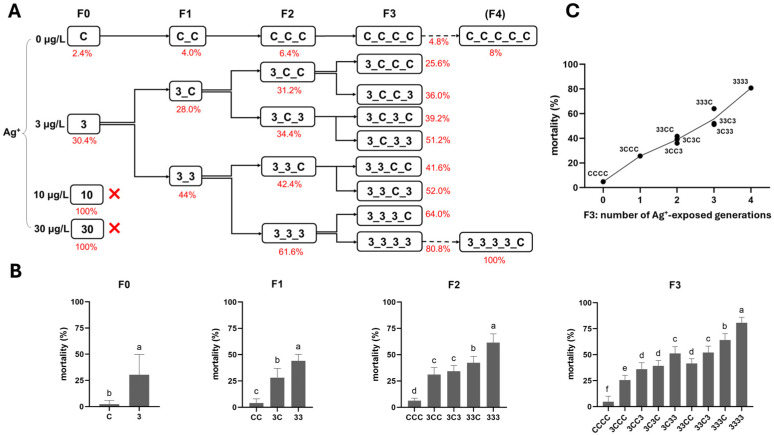
The effect of Ag^+^ (nominal concentrations 0, 3, 10, and 30 µg/L) on the mortality of *Chironomus riparius* across four generations (n = 5). (**A**) Mortality rates (means, in red) across generations in the conceptual diagram of Experiment 1 (both 10 and 30 µg/L Ag^+^ groups showed 100% mortality at F0, preventing further generational studies). (**B**) Mortality rates in each generation, means ± standard deviations. Different letters above the columns indicate significant differences (*p* < 0.05) between groups within each generation (F0: Mann–Whitney U test; F1–F3: ANOVA, LSD test). (**C**) Mean mortality rates of *C. riparius* in F3 vs. number of Ag^+^-exposed generations, independent of exposure history.

By the end of the first-generation (F0) study, the control group and the 3 µg/L Ag^+^ treatment group exhibited low and acceptable mortality rates, respectively. However, complete mortality was observed in the 10 and 30 µg/L Ag^+^ treatment groups (i.e., “10” and “30”), preventing further experimentation at these two concentrations. Consequently, only the control and the 3 µg/L Ag^+^ treatment groups (i.e., “C” and “3”) were continued for subsequent generations (Figure 1 and Figure 2). For the continuation of this multi-and trans-generational study, offspring from the first generation of the 3 µg/L Ag^+^ treatment group were randomly divided into two groups: one group was re-exposed to the test solution of 3 µg/L Ag^+^ (named “33”), while the other was transferred to a clean medium (named “3C”). This exposure scheme was designed for all of the four generations, resulting in a total of 15 test groups (1× F0 + 2× F1 + 4× F2 + 8× F3) plus 4 control groups (1× F0, F1, F2, F3) (Figure 1). After four generations of 3 µg/L exposure, however, even transfer to clean water resulted in 100% mortality, preventing further generational studies.

### 2.4. Experiment 2: Simulation of Contamination Scenarios with Multiple Generations of Continuous Exposure to Low Concentrations of Ag^+^ and Subsequent Recovery

Experiment 2 was designed to investigate long-term Ag^+^ effects on *C. riparius* across seven successive generations. It included three environmentally relevant Ag^+^ concentrations (nominal 0.01, 0.1 and 1 µg/L) in addition to the 3 µg/L level from Experiment 1, plus a control (Figure 1). In the first phase (multigenerational exposure), four consecutive generations (F0–F3) were continuously exposed to each concentration. In the second phase (transgenerational recovery), populations that had been exposed for multiple generations were subsequently reared in clean water for three generations (testing recovery in F1′–F3′). Experiment 2 employed the same operational procedures and conditions as Experiment 1 except for the experimental design. This two-phase approach aimed at providing more realistic insights into the long-term ecological risks of Ag^+^.

### 2.5. Endpoints in Chronic Toxicity Tests of Ag^+^

During the experiments, numerous life-traits were measured/calculated, mostly following the OECD guidelines [39]. The parameters mortality and emergence rate were recorded in each replicate. Mortality was determined by counting the number of surviving larvae, pupae, and emerged adults in each replicate throughout the exposure period of each generation. Mortality in long-term studies was expressed as the proportion of dead individuals relative to the initial 25 first instar larvae introduced per replicate. Individuals that did not develop to the next life stage (e.g., failed larval or pupal development, unsuccessful emergence) were also considered dead. The emergence rate was calculated as the ratio between the total number of emerged adults and the number of the larvae inserted at the beginning of the test. Reproduction parameters comprised fertility, calculated by dividing the total number of fertile egg ropes in each cage by total number of adult females added to each breeding cage (egg ropes are kept for observation for at least six days after they have been produced so that they can be classified as fertile or infertile. An egg rope is considered fertile when at least one third of the eggs hatch.); the average number of eggs per egg rope, determined according to the ring counting method [36]: the number of eggs from the middle spiral was multiplied with the number of spirals per egg rope when the egg ropes present a normal C-shape; the percentage of females, calculated as the number of adult females divided by the total numbers of emerged adults. Regarding growth parameters, dead adults (7 males and 7 females) were collected separately and dried at 50 °C for 48 h to determine the average dry body weight. Since *C. riparius* has a bimodal emergence pattern where males emerge prior to females [61], the development rate and mean emergence time (Emt50; the day that half of the new generation emerged) were calculated separately for each sex.

Finally, all these parameters were combined to monitor overall population fitness by calculating the population growth rate (PGR), an integrating endpoint in life-cycle experiments. The PGR is sensitive to the negative effects of pollutants on population growth levels [50]. The parameter represents the potential growth capacity of a population. Thus, to evaluate the potential effects of Ag^+^ on the population level based on the considered life-traits, the PGR was calculated using the following equation [62]:

PGR = [(*e* × *d* × *c*) × (1 − *a*/100)]^1/*b*^

where *e* is the number of fertile egg ropes per female; *d* is the average number of eggs per egg rope; *c* is the female fraction on total individuals emerged; *a* is the percentage of mortality; and *b* is the mean development time of females.

### 2.6. Data Analysis

Normality of data was checked with the Shapiro-Wilk (S-W) test, and homogeneity of variances with Levene’s test. To further evaluate the significance of effects, according to the number of pairs, the normality of the data and the homogeneity of the variance across the treatment groups, the following statistical tests were used: Student’s *t*-test, linear regression analysis, one-way analysis of variance (ANOVA) followed by Fisher’s least significant difference (LSD) multiple comparisons, or nonparametric tests, including Mann–Whitney U test and Kruskal–Wallis test, followed by Bonferroni correction. All statistical analyses were performed using SPSS 22.0. For all statistical tests, the level of significance was considered as *p* < 0.05.

## 3. Results

### 3.1. Experiment 1: Multiple Generations Exposed to Pulses of Contamination and to Recovery Phases

#### 3.1.1. Survival (Mortality and Emergence)

After the F0 generation exposure in Experiment 1, 100% mortality occurred in the 10 and 30 µg/L Ag^+^ groups, whereas the 3 µg/L group (the only surviving exposure group besides the control) showed acceptably low mortality, allowing the test to continue (Figure 2).

**Figure 3:**
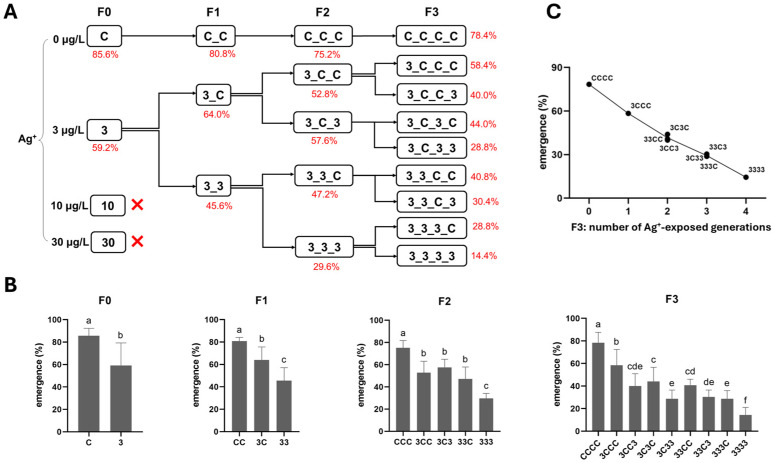
The effect of Ag^+^ (nominal concentrations 0, 3, 10, and 30 µg/L) on the total emergence of *Chironomus riparius* across four generations (n = 5). (**A**) Emergence rates (means, in red) across generations in the conceptual diagram of Experiment 1 (both 10 and 30 µg/L Ag^+^ groups showed 0% emergence at F0, preventing further generational studies). (**B**) Emergence rates in each generation, means ± standard deviations. Different letters above the columns indicate significant differences (*p* < 0.05) between groups within each generation (F0: Mann–Whitney U test; F1–F3: ANOVA, LSD test). (**C**) Mean emergence rates of *C. riparius* in F3 vs. number of Ag^+^-exposed generations, independent of exposure history.

Here, multi- and transgenerational Ag^+^ toxicity effects were found for both mortality and emergence rate. First, along the exposure routes 3–33–333–3333, both endpoints worsened generation by generation (higher mortality, lower emergence; Figure 2 and Figure 3). Second, whether exposure occurred in a single generation or across successive generations, when the next generation was reared in clean water, the recovery lines (i.e., 3C, 33C and 333C) displayed levels of both survival endpoints comparable to those of their respective parental exposure groups in the previous generation (i.e., 3 vs. 3C, 33 vs. 33C, and 333 vs. 333C; Figure 2 and Figure 3). However, within the same generation, both survival endpoints of exposed groups performed significantly worse than their corresponding recovery groups (33 vs. 3C, 333 vs. 33C, and 3333 vs. 333C; Figure 2 and Figure 3). By the end of the experiment (F3 generation), both mortality and emergence depended only on the total number of generations exposed to Ag^+^, regardless of which specific generations were exposed or not. In other words, whether the parents, grandparents, or great-grandparents had been exposed made no difference—the cumulative number of exposure generations was the determining factor (Figure 2 and Figure 3). Furthermore, significant transgenerational effects of the 3 µg/L Ag^+^ treatment were also evident. Mortality and emergence impairment persisted in the recovery lineages, including 3–3C–3CC–3CCC, 33–33C–33CC, and 333–333C (Figure 2 and Figure 3).

#### 3.1.2. Development (Emt50 and Development Rate)

In the first two generations (F0–F1), the 3 µg/L Ag^+^ treatment groups showed significantly delayed Emt50 compared to respective control groups (i.e., 3 vs. C and 33 vs. CC) in both males and females (Figure S1), even though the effect size was rather small. In addition, also a transient transgenerational (3–3C) effect of 3 µg/L Ag^+^ on Emt50 was found in females (F0–F1 in Figure S1). However, in later generations (F2–F3), no significant differences in Emt50 were observed between continuously exposed and control groups (i.e., 333 vs. CCC and 3333 vs. CCCC) for both males and females (Figure S1).

Similar results were recorded for the development rate. In generation F1, the male development rate in the continuous 3 µg/L group (33) was significantly lower than in the control (i.e., CC, Figure S2). For females, the continuous 3 µg/L groups in F0 and F1 (i.e., 3 and 33) exhibited significantly lowered development rate compared to the respective control groups (i.e., C and CC, Figure S2). In addition, transient transgenerational (3–3C) and multigenerational (3–33) effects of 3 µg/L Ag^+^ on the development rate were also found in females (F0–F1 in Figure S2). By F2–F3, development rates in continuously exposed groups were not significantly different from controls for either sex (i.e., 333 vs. CCC and 3333 vs. CCCC; Figure S2).

In summary, the development-related endpoints (Emt50 and development rate) were less sensitive to 3 µg/L Ag^+^ exposure than survival-related endpoints (mortality and emergence). Any multigenerational effects on development occurred mainly in the first two generations, and females showed somewhat higher susceptibility than males.

#### 3.1.3. Reproduction (Fertility, Average Egg Numbers per Egg Rope and Female Percentage) and Growth (Based on Dry Body Weight)

**Figure 4:**
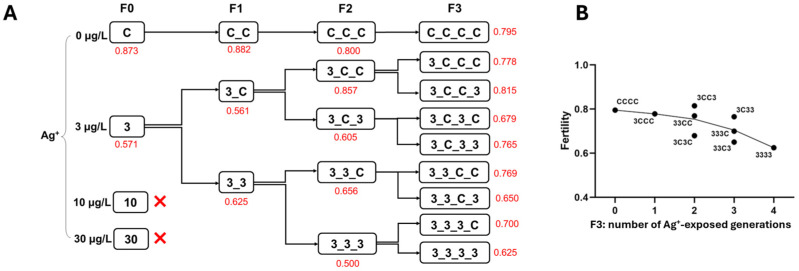
The effect of Ag^+^ (nominal concentrations 0, 3, 10, and 30 µg/L) on the fertility of *Chironomus riparius* across four generations. (**A**) Fertility values (in red) across generations in the conceptual diagram of Experiment 1 (both 10 and 30 µg/L Ag^+^ groups did not produce adults). The fertility of each treatment group is given as a single value since, for each generation, all adults from 5 replicates of each treatment were collected in a single breeding cage. (**B**) Fertility values of *C. riparius* in F3 vs. number of Ag^+^-exposed generations, independent of exposure history.

Fertility exhibited a declining trend across generations at 3 µg/L Ag^+^ (Figure 4). In F0, the 3 µg/L group produced significantly fewer eggs per rope than the control group. However, in F1–F3, no significant differences in the egg number per rope were observed between the continuous 3 µg/L Ag^+^ groups and the respective control groups (i.e., 33 vs. 3C, 333 vs. CCC and 3333 vs. CCCC; Figure S3). Female emergence ratios did not show significant differences among treatment groups across four generations except in the F2 generation, where the Ag^+^-exposed group had a lower proportion of females compared to the control group (Figure S4).

No significant differences in adult dry body weight were detected between treated and control groups for either sex across all generations (Figure S5). In general, females were a little heavier than males.

#### 3.1.4. Population Growth Rate (PGR)

**Figure 5:**
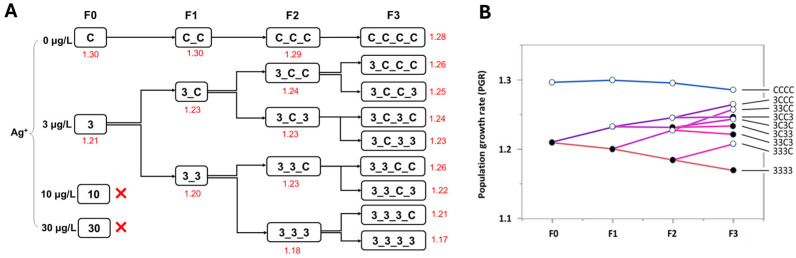
The effect of Ag^+^ (nominal concentrations 0, 3, 10, and 30 µg/L) on the population growth rate (PGR) of *Chironomus riparius* across four generations. (**A**) PGRs (in red) across generations in the conceptual diagram of Experiment 1 (in both 10 and 30 µg/L Ag^+^ groups populations became extinct). (**B**) PGRs of *C. riparius* during the course of a series of different contamination pulses. Black dots: current population Ag+-exposed, circles: current population non-exposed.

The PGR declined with increasing number of generations exposed to 3 µg/L Ag^+^, although in all cases PGR remained above the critical value of 1. Each successive exposed generation had a lower PGR than its corresponding control. As the number of ‘recovery’ generations increased, the PGR of the great-grandchildren of the Ag^+^-exposed F0 generation approached that of the CCCC control group (Figure 5).

### 3.2. Experiment 2: Simulation of Contamination Scenarios with Multiple Generations of Continuous Exposure and Subsequent Recovery

#### 3.2.1. Survival (Mortality and Emergence)

**Figure 6:**
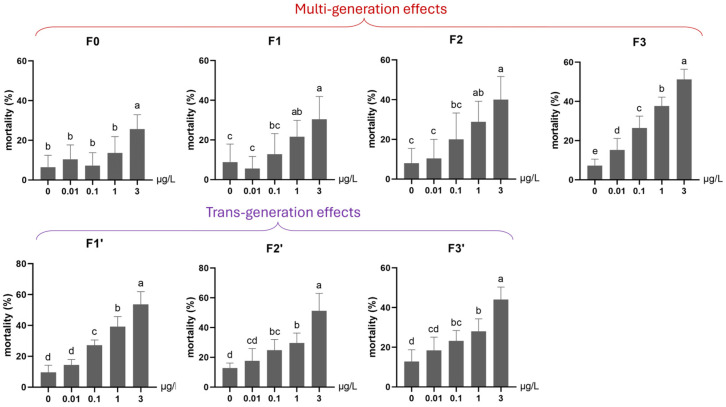
The effect of Ag^+^ (nominal concentrations 0, 0.01, 0.1, 1 and 3 µg/L) on the mortality of *Chironomus riparius* across seven successive generations (n = 5), including multigenerational exposure of the first four generations (F0–F3) and transgenerational recovery of the subsequent three generations (F1′–F3′). Means ± standard deviations. Different letters above the columns indicate significant differences (*p* < 0.05) between groups within each generation (F0–F3 and F1′–F3′: ANOVA, LSD test).

**Figure 7:**
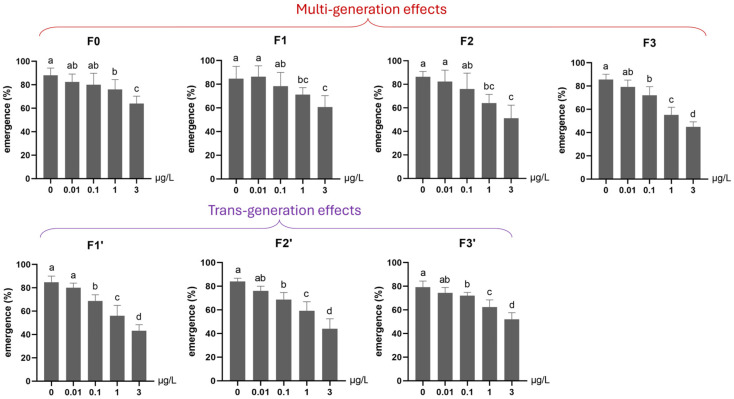
The effect of Ag^+^ (nominal concentrations 0, 0.01, 0.1, 1 and 3 µg/L) on the emergence of *Chironomus riparius* across seven successive generations (n = 5), including multigenerational exposure of the first four generations (F0–F3) and transgenerational recovery of the subsequent three generations (F1′–F3′). Means ± standard deviations. Different letters above the columns indicate significant differences (*p* < 0.05) between groups within each generation (F0–F3 and F1′–F3′: ANOVA, LSD test).

Under continuous low-level exposure (Experiment 2), significant multigenerational adverse effects were observed on mortality across all Ag^+^ treatment groups (0.01, 0.1, 1, 3 μg/L) throughout four generations (F0–F3; Figure 6). Particularly, a significantly elevated mortality rate was found for an Ag^+^ concentration as low as the EU-EQS of 0.01 μg/L [23,27] in the 4th generation (F3, Figure 6). The emergence rate was significantly reduced in the first (F0) and the following Ag^+^-exposed generations by ≥1 µg/L and in the fourth generation (F3) by ≥0.1 µg/L (Figure 7).

During the subsequent recovery phase (F1′–F3′), transgenerational effects on both survival parameters persisted in the 0.1, 1, and 3 µg/L groups. Elevated mortality and depressed emergence remained detectable through the final recovery generation (F3′) in these lineages (Figure 6 and Figure 7).

#### 3.2.2. Development (Emt50 and Development Rate)

Multigenerational effects on Emt50 were intermittent with these effects only occurring on certain occasions. In males, the 3 µg/L Ag^+^ group showed significantly delayed Emt50 in three of the four exposed generations (F0, F2 and F3), and a minor delay at 1 µg/L occurred in F2, and the effect size was generally small. During the recovery phase (F1′–F3′), the Emt50 of males delays re-occurred in the 1 and 3 µg/L Ag^+^ lineages by F3′ (Figure S6). In females, sustained significant multigenerational delays were observed in the 1 and 3 µg/L Ag^+^ groups across F0–F3, while the 0.1 µg/L Ag^+^ treatment group showed a significant delay just in F1. Transgenerational delays in female Emt50 persisted in the 1 and 3 µg/L groups throughout the three recovery generations (F1′–F3′) (Figure S6).

Development rate showed clearer sustained effects. In males, the 3 µg/L groups showed a significantly slower development rate in two of the four exposure generations (F0 and F2) compared to the respective control groups. Subsequently, transgenerational effects at 3 µg/L Ag^+^ persisted, with reduced male development rates in all three recovery generations (F1′–F3′) (Figure S7). In females, the 1 and 3 µg/L Ag^+^ groups had a consistently lower development rate across F0–F3. During the subsequent recovery phase, both the 1 and 3 µg/L lineages continued to exhibit significantly reduced female development rates in each recovery generation (F1′–F3′), indicating persistent transgenerational effects of Ag^+^ (Figure S7).

#### 3.2.3. Reproduction (Fertility, Average Egg Numbers per Egg Rope and Female Percentage) and Growth (Based on Dry Body Weight)

**Figure 8:**
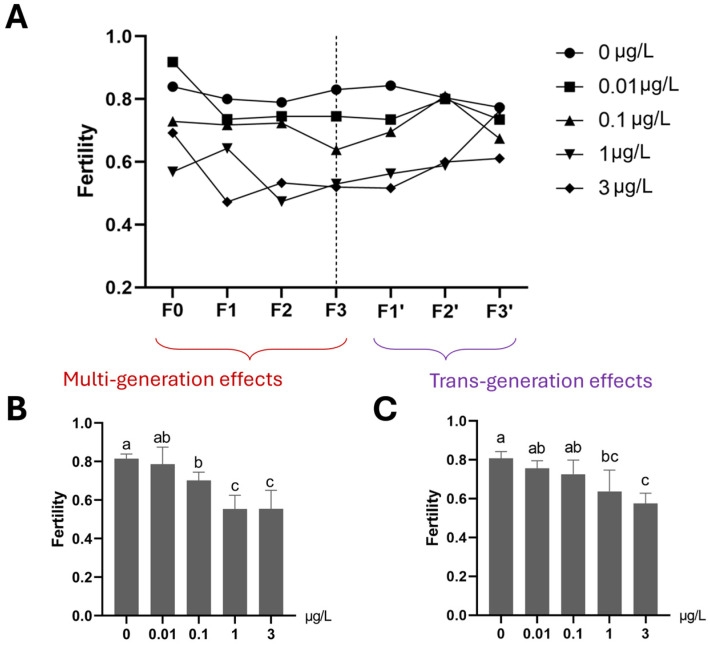
The effect of Ag^+^ (nominal concentrations 0, 0.01, 0.1, 1 and 3 µg/L) on fertility of *Chironomus riparius* across seven generations, including multigenerational exposure of the first four generations (F0–F3) and transgenerational recovery of the subsequent three generations (F1′–F3′). (**A**) Fertility patterns in different Ag^+^ exposure regimes across seven generations. The fertility of each treatment group is given as a single value since, for each generation, all adults from 5 replicates of each treatment were collected in a single breeding cage. (**B**) The average fertility of each concentration treatment for the first four, Ag^+^-exposed generations (F0–F3, n = 4). (**C**) The average fertility of each concentration treatment for the subsequent three recovery generations (F1′–F3′, n = 3). For (**B**) and (**C**) data are expressed as means ± standard deviations. Different letters above the columns indicate significant differences between groups (ANOVA, LSD test: *p* < 0.05).

**Figure 9:**
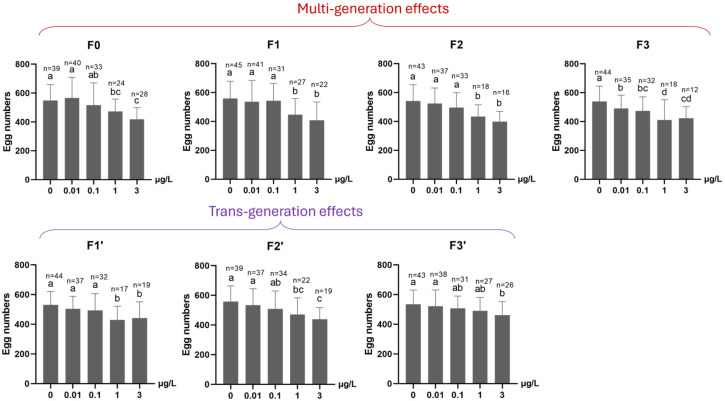
The effect of Ag^+^ (nominal concentrations 0, 0.01, 0.1, 1 and 3 µg/L) on the average number of eggs per egg rope of *Chironomus riparius* across seven successive generations (n number given above the columns), including multigenerational exposure of the first four generations (F0–F3) and transgenerational recovery of the subsequent three generations (F1′–F3′). Means ± standard deviations. Different letters above the columns indicate significant differences (*p* < 0.05) between groups within each generation (F0: Kruskal–Wallis test plus Bonferroni correction; F1–F3 and F1′–F3′: ANOVA, LSD test).

Mean fertility in each of the 0.1, 1 and 3 µg/L Ag^+^ treatment groups was significantly decreased compared to the control group if the average of all exposed generations in the F0–F3 period is considered (Figure 8). For the subsequent three-generation recovery period (F1′–F3′), transgenerational effects persisted, on average, in both the 1 and 3 µg/L Ag^+^ treatment groups until the final recovery generation (F3′), although fertility rates converged at the end of the recovery period. (Figure 8). The average number of eggs per egg rope was found to be reduced in all ≥1 µg/L-exposed generations and all their subsequent “recovery” generations (Figure 9). In the fourth Ag^+^-exposed generation, F3, a significant effect on this parameter was evident even at 0.01 µg/L Ag^+^ (the EU-EQS level) [23,27], and at all higher concentrations (Figure 9).

Hardly any or no significant differences (at the *p* = 0.05 level) were found among all treatment groups for both the rate of females (Figure S8) and the dry body weight of females and males (Figure S9) in any generation (F0–F3′).

#### 3.2.4. Population Growth Rate (PGR)

**Figure 10:**
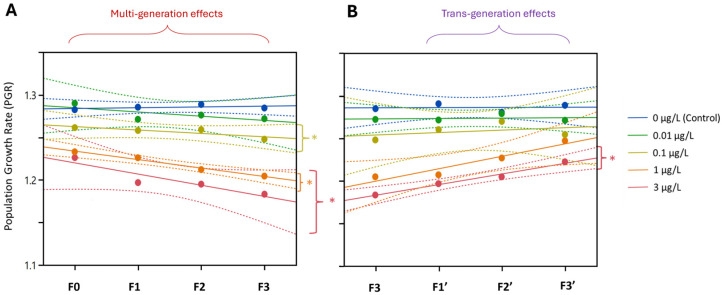
The effect of Ag^+^ (nominal concentrations 0, 0.01, 0.1, 1 and 3 µg/L) on the population growth rate (PGR) of *Chironomus riparius* across seven successive generations including (**A**) multigenerational exposure of the first four generations (F0–F3) and (**B**) transgenerational recovery of the subsequent three generations (F1′–F3′). Linear regression lines and 95% confidence intervals. Asterisks indicate those exposures for which the 95% confidence intervals in the positions of the last generations (F3, F3′) did not overlap with those of the controls.

After four generations of continuous exposure, PGR was significantly reduced at concentrations ≥ 0.1 µg/L Ag^+^ (Figure 10). This reduction persisted through the three recovery generations in the lineage previously exposed to 3 µg/L Ag^+^, whereas the lineages from ≤ 1 µg/L exposures showed PGR increases approaching control levels. In all cases, PGR remained >1 for all exposed and recovery generations.

## 4. Discussion

Ag^+^ exhibits low chronic toxicity thresholds across diverse aquatic trophic levels [23]. In this study, we systematically evaluated the chronic toxicity of Ag^+^ to *C. riparius* using two experimental frameworks. Our results provide critical insights into its long-term ecological risks even at very low concentrations under favorable conditions (optimal temperature, sufficient food, absence of predators as well as no other contaminants). This work underscores the need to incorporate multigenerational and transgenerational perspectives into ecotoxicological assessments, especially for persistent contaminants like Ag^+^, where sublethal effects can persist or even amplify over generations.

Experiment 1 demonstrated that exposure to concentrations selected from acute toxicity tests (10 and 30 µg/L Ag^+^) resulted in complete mortality by the end of the first generation (F0). Only *C. riparius* that were exposed to 3 µg/L of Ag^+^ (one-tenth of the EC_10_ value) were able to reproduce successfully, with subsequent generations continuing to do so. Experiment 2 further revealed that chronic exposure to much lower Ag^+^ concentrations (down to the EU-EQS of 0.01 µg/L) [27] can produce clear population-level impacts. These findings directly confirm that concentrations causing chronic multigenerational effects are far below those causing acute toxicity, consistent with previous studies [63]. Our study also reinforces that time is widely considered to be an important factor linking environmental exposure to individual and population effects [64,65,66]. Even very low chronic Ag^+^ inputs can depress PGR, posing a serious threat to *C. riparius* populations, especially when combined with other stressors (e.g., climate, food limitation, predation). This supports concerns that reliance on acute toxicity data may underestimate risk and lead to insufficiently protective standards [1,67]. The current lack of chronic Ag toxicity data contributes to the potentially insufficient stringency of environmental quality standards for Ag in natural waters [67,68].

### 4.1. Experiment 1

In Experiment 1, continuous and pulsed exposure at 3 µg/L Ag^+^ induced significant, cumulative adverse effects on survival-related parameters (mortality and emergence) across generations. We also observed population-level effects in *C. riparius*, which is generally considered highly tolerant to environmental stress, with extinction by the fifth generation at 3 µg/L Ag^+^. The greater the number of generations exposed to Ag^+^, the stronger the multigenerational effect was, regardless of which generations were exposed. Exposure of ancestors had a similar effect as the current generation’s own exposure. These highlights mentioned above are supported by the following findings: continuous exposure lineages exhibited progressively higher mortality and lower emergence with each generation (e.g., 3 vs. 3C, 33 vs. 33C and 333 vs. 333C), and recovery lineages (with intermittent clean generations) showed mortality and emergence rates nearly as poor as their continuously exposed counterparts (e.g., 33 vs. 3C, 333 vs. 33C and 3333 vs. 333C). At 3 µg/L Ag^+^, progressive decline in survival and emergence with advancing generations under continuous exposure of Ag^+^ (3–33–333–3333). In addition, at 3 µg/L Ag^+^, general trend towards lower emergence rates and increased mortality by the final generation (F3), correlating with the total number of generations exposed regardless of the specific pulse history.

Reproductive parameters (fertility) and the PGR showed similar patterns of decline at 3 µg/L Ag^+^, indicating clear transgenerational toxicity. Developmental endpoints (Emt50 and development rate) showed moderate sensitivity to 3 µg/L Ag^+^ exposure compared to survival endpoints: only unstable effects appeared in the first two generations, with females appearing more susceptible than males. Adult dry weight and the female emergence proportion were unaffected by 3 µg/L Ag^+^ exposure.

*Chironomus riparius* is recognized as an important indicator species for metal bioaccumulation [40,41,42]. The uptake of ionic Ag from the water phase leads to intracellular silver deposits which persist during molting. The accumulation of Ag^+^ in adult *C. riparius* has been discussed as a warning sign of chronic impairment [69]. Also in earthworms, *Eisenia fetida,* exposed to ionic silver, the toxicity level in reproduction was correlated with internal concentration [70]. Moreover, a long-term Ag^+^ exposure study on the soil arthropod *Folsomia candida* revealed high bioaccumulation and associated high mortality and reproductive toxicity. Dose–response relationships were observed for the effect of Ag^+^ on the survival and reproduction of this species [71]. These findings, combined with our results, suggest that once Ag^+^ accumulation and toxicity occur in *C. riparius*, the damage is persistent and cumulative across generations, particularly in highly contaminated environments, rather than fluctuating strongly with pulse history. This aligns with the characteristics of environmental chemicals of concern: bioaccumulation, persistence, and toxicity [67]. Comparable trends include increasing mortality and decreasing population growth in *Daphnia magna* over five generations of AgNP exposure [72] and the rapid deterioration of survival in *C. riparius* under multigenerational sublethal exposure to Cadmium (Cd) [73]. Moreover, severe mortality of *C. riparius* exposed to tributyltin over multiple generations increases with number of generations exposed [36]. Furthermore, chronic exposure to the Benzo(a)pyrene (BaP) over three generations significantly reduced *C. riparius* population growth rate, with no signs of rapid adaptation [37]. In addition, many multigenerational studies on *C. riparius* have reported that F1 generations are more affected than F0 generations by exposure to pyriproxyfen and teflubenzuron [74,75]. Similarly, the F1 and F2 offspring of *C. riparius* exposed to the insecticide fipronil (alone or with 2,4-D) showed greater mortality and reduced emergence compared to the parental generation [58]. In all these studies, including ours, there was no sign of microevolutionary adaptation within those first generations that can be decisive for the persistence of the population in the event of environmental contamination. The progressive worsening in survival, development, and reproduction across generations (3–33–333–3333) showed no evidence for a genetic adaptation to 3 µg/L Ag^+^. Recovery lineages from both single and multigenerational exposure at this concentration consistently showed significantly elevated mortality and reduced emergence (e.g., 3–3C–3CC–3CCC; 33–33C–33CC and 333–333C), indicating persistent transgenerational toxicity on survival. A comparable phenomenon has also been demonstrated for *C. riparius* exposed to the organic UV-filter benzophenone-3 (BP3): after only parental exposure, F1 reared in uncontaminated media still showed reduced emergence and altered development time, evidencing transgenerational impairment despite the return to clean conditions [76]. In addition, studies have revealed that exposure to endocrine disruptors can cause transgenerational epigenetic effects, as shown in *Daphnia magna* [77,78], *Aedes albopictus* [79], and vertebrates [80]. Considering that Ag is also known to act as an endocrine disruptor [81], and endocrine disruption has been linked to heritable epigenetic effects in invertebrates [77,78,79,80]. this suggests that the transgenerational effects on reproduction relevant parameters observed at 3 µg/L Ag^+^ may also involve epigenetic mechanisms. Thus, epigenetic mechanisms may involve the transgenerational reproductive impairments observed at 3 µg/L Ag^+^ in our experiment.

The severe multigenerational impairment of survival and emergence resulted in reduced fertility and a decline in PGR. Although the PGR remained above the critical threshold of 1 in all groups by the end of experiment 1, indicating no immediate population collapse [50] in the current experiment, chronic exposure to 3 µg/L Ag^+^ is still alarming for long-term population stability, especially under realistic field conditions where additional stressors are present. This conclusion is reasonable, as individual survival and emergence are prerequisites for reproduction, the key link between individuals and populations [75,82,83,84]. Furthermore, the PGR measured for lab controls must be regarded as merely population-preserving under natural conditions. Impaired reproduction parameter (fertility) adversely impacts populations [83], as previously reported in *C. riparius* for metals, organometals [62,85], and pharmaceuticals [86]. Our findings of sustained fertility impairment emphasize its importance as a sensitive endpoint in life-cycle tests.

Regarding development parameters, transient negative transgenerational and multigenerational effects were only found in females during early two generations (e.g., 3–33 and 3–3C), indicating sex-specificity of Ag^+^ action in this respect. Similar sex-specific developmental sensitivity in *C. riparius* has been reported for Cd [87]. Differential impacts on male and female development can disrupt mating synchrony, adversely affecting reproduction and population dynamics [76]. On the other hand, growth parameters (dry body weight) [48] and the female ratio of the emerged individuals remained unaffected, which can reduce impacts on reproduction. In our study, the normal growth and balanced sex ratio of *C. riparius* could help to alleviate further impairment to reproduction caused by the continuous damage to survival, emergence and fertility, as well as changes in male-female synchrony resulting from sex-specific sensitivity differences.

Multigenerational exposure to persistent chemicals at sublethal, environmentally relevant concentrations is essential for robust risk assessment [88,89], as in the case of silver in our study. While a concentration of 3 µg/L Ag^+^ in our study is environmentally relevant [20], it may exceed most environmental levels but rather represents heavily contaminated industrial discharge zones, such as sewage outfalls, electroplating plants, and mine waste dumps [21]. More commonly, long-term exposure to chemicals occurs at persistent lower concentrations [49]. In order to establish a more comprehensive understanding of the chronic toxic effects of Ag^+^ on *C. riparius* at common lower environmentally relevant concentrations and its potential for recovery, we have designed Experiment 2.

### 4.2. Experiment 2

Experiment 2 largely corroborated the trends seen in Experiment 1 while extending the assessment to lower concentrations and through recovery generations. The 3 µg/L exposure in Experiment 2 produced similar survival-related patterns (a continuous worsening trend as the number of exposed generations has increased). However, the impairment of *C. riparius* under the 3 µg/L Ag^+^ exposure condition was generally less severe per generation in Experiment 2 than in Experiment 1. This likely reflects random genetic diversity between initial larval batches, a natural variation arising from routine mixing of laboratory breeding cages to maintain genetic health. This phenomenon has been observed in other long-term multigenerational studies, where genetic variation among strains of *C. riparius* from different laboratories resulted in different sensitivity levels to sublethal low Cd concentrations [50,59], indicating that such variability is a normal and reasonable occurrence in laboratory experiments. The lower mortality in Experiment 2 actually facilitated a more detailed evaluation of sublethal endpoints (development, reproduction, etc.), which might be obscured under more extreme mortality, such as reduced energy allocation to offspring by stressed parents [76] producing offsprings with less fitness [90]. Therefore, based on the results from Experiment 1, Experiment 2 further provides a more detailed and robust assessment of long-term Ag^+^ impacts on *C. riparius* fitness.

The key findings from Experiment 2 were the following: We observed multigenerational effects in life history parameters (survival, development, reproduction and population stability) at levels that were either equal to or near international environmental quality standards for freshwater [23]. A significant reduction in the population growth rate occurred at 0.1 µg/L Ag^+^ (10 × EU-EQS) [27]. Experiment 2 also showed no evidence of a selection of tolerant genotypes after four generations of Ag^+^ exposure, even under strong selection pressure. Transgenerational effects were evident for many endpoints as well, although a gradual recovery trend appeared over three unexposed generations.

The summary of multigenerational effects of Ag^+^ on *C. riparius* mentioned above is supported by the following detailed observations: In the Ag^+^-exposed generations F0–F3, significant cumulative toxicity on survival parameters (mortality and emergence) occurred at 0.1, 1, and 3 µg/L Ag^+^. In particular, when compared with the current international list of available threshold values for Ag in freshwater environments, we found that elevated mortality in the fourth generation (F3) indicated that a nominal concentration of 0.01 µg/L of Ag^+^ impacted *C. riparius*, reaching the potential implementation level for silver in the EU-EQS and falling below but close to the standards of Denmark (0.017 µg/L), Germany (0.02 µg/L) and Australia (0.05 µg/L) [20]. Regarding developmental parameters (Emt50 and development rate) in the generations F0–F3, females exhibited significant, stable delays in Emt50 and a decreased development rate at 1 and 3 µg/L Ag^+^ across generations. Developmental endpoints showed a clear sex-specific pattern: females had consistently delayed Emt50 and reduced development rates at 1 and 3 µg/L across generations (with a transient delay at 0.1 µg/L in F1), whereas males showed significant developmental impairment only at 3 µg/L Ag^+^ (delayed Emt50 in F0, F2, F3; decreased development rate in F0, F2). Combined with the findings of Experiment 1, Experiment 2 further confirmed the higher female sensitivity to Ag^+^ mirroring the sex-specific toxicity pattern of Cd in *C. riparius* [87]. Significant multigenerational declines of reproductive output declined markedly at 1 and 3 µg/L—both fertility and average egg number per rope were reduced. By F3 even the 0.01 (EU-EQS level) [23,27] and 0.1 µg/L groups produced significantly fewer eggs per rope, indicating that chronic parental stress at sublethal levels may reduce offspring fitness evidently [90]. In contrast, dry body weight of adults remained unaffected. Undamaged growth is beneficial to reproductive success to some extent, which alleviates the impact of impairment of other reproduction-related parameters and hence the stability of the population [48]. Although the PGR remained >1 after four generations, significant declining trends occurred at concentrations ≥0.1 µg/L Ag^+^. Considering that the Ag^+^ contamination in the real environment would be more persistent rather than just the four generations covered in this study, prolonged exposure beyond four generations at ≥0.1 µg/L Ag^+^ could threaten population stability of *C. riparius*. This notion is supported by multigenerational tests with other contaminants: for example, chronic low-dose Cd exposure in *C. riparius* produced detectable impacts only after several successive generations, underscoring the need for extended exposure durations to reveal such latent effects [50]. Given that Ag^+^ is not the only stress factor under natural environmental conditions, this perspective is particularly important. Furthermore, regarding transgenerational effects in the ‘recovery’ generations (F1′–F3′), the offspring from 0.01 µg/L Ag^+^ exposure recovered and reached normal mortality by F1′ and normal emergence by F2′. Significant transgenerational adverse effects of these two parameters persisted throughout the three recovery generations at concentrations of 0.1, 1, and 3 µg/L Ag^+^. Transgenerational developmental toxicity (delayed Emt50, reduced development rate) persisted for both sexes from the 1 and 3 µg/L Ag^+^ lineages, with effects being more stable in females. Reduced fertility persisted in F3′ offspring from 1 and 3 µg/L Ag^+^. Concerning the transgenerational effects of Ag^+^ on reproduction-related parameters (fertility and average egg numbers per egg rope), the fertility of the recovered offspring at 1 and 3 μg/L Ag^+^ remained significantly reduced at the end of F3′. However, the most sensitive reproductive parameter, i.e., the average egg numbers per egg rope, recovered in F1′ for the 0.01 and 0.1 µg/L Ag^+^ groups, and by the F3′ generation for the 1 µg/L Ag^+^ group, but remained significantly depressed in the 3 µg/L Ag^+^ group even at F3′. It is possible that the PGR will continue to be lower than that of the control group also in later generations.

The results of the “recovery” phase showed that the effects of low concentrations (<0.1 µg/L Ag^+^) were reversed quickly within one recovery generation (F1′), which suggests that the mechanisms involved are mainly physiological (e.g., metallothionein induction oxidative stress responses [91]), rather than genetic. Similar toxicity response patterns and explanations have also been reported in multi- and trans-generational studies on mercury in *Tigriopus japonicus* [92]. In contrast, environmentally relevant concentrations of Ag^+^ (≥0.1 µg/L) at relatively high sublethal levels for *C. riparius* induced more sustained transgenerational toxicity, with longer persistence, correlating with higher concentration. The sustained effects at 3 µg/L Ag^+^ even after three recovery generations (F3′) suggest the induction of mechanisms involving heritable changes, preventing rapid reversal in the following generations. Similar persistent transgenerational effects linked to epigenetics occurred in the nematode *Caenorhabditis elegans* exposed to Ag nanomaterials [93]. Our findings thus also may involve epigenetic mechanisms (e.g., DNA methylation) underlying persistent transgenerational toxicity at high Ag^+^ concentrations, that are heritable but also reversible and across generations without modifying gene sequences [94]. Further, inspired by this, targeted assays (e.g., methylation profiling) should be pursued in future work.

Overall, long-term, multigenerational Ag^+^ exposure in contaminated rivers persistently harms individual survival, development, and reproduction in *C. riparius*. The population stability relevant parameter, PGR, indicates potential disruption of natural populations under chronic contamination. Generally, most sensitive for Ag^+^ were survival-related parameters (mortality and emergence). Crucially, the observed long-term survival and reproductive toxicity at a concentration of 0.01 µg/L of Ag^+^ raises concerns regarding the adequacy of current threshold values for freshwaters in the EU-EQS [23,27]. Furthermore, even after three generations of recovery following the four consecutive generations of exposure to Ag^+^ at different concentrations, we also observed transgenerational impairment on survival, development, reproduction, and population growth, of which transgenerational survival toxicity was most evident. This justifies selecting mortality and emergence as the two most critical endpoints for investigating the long-term toxicity of contaminants to *C. riparius*, rather than considering other life history traits. Our results further emphasize the necessity of adopting multigenerational endpoints into regulatory frameworks, especially for highly persistent and bioaccumulative pollutants like Ag. It is worth noting that, exposures in this study were conducted using nominal concentrations, which is standard practice in long-term *Chironomus riparius* assays to ensure comparability across studies. Nonetheless, considering that the stability of Ag^+^ in aqueous media is subject to alteration through precipitation, complexation, and redox or photochemical processes [95,96,97]. While analytically measured concentrations were not determined here, their inclusion would provide a more precise characterization of exposure conditions. This limitation is recognized and will be addressed in future investigations to further strengthen methodological robustness.

Ecologically, as the most abundant benthic insect group, *C. riparius* larvae are a crucial food source in aquatic ecosystems, while adults connect aquatic and terrestrial food webs [98]. The most important route of Ag^+^ uptake for *C. riparius* is from water. Silver can accumulate efficiently in *C. riparius* and has high biomagnification potential [69]. Adults can thus act as vectors transferring Ag^+^ from aquatic to terrestrial ecosystems [99]. Our results suggest this transfer to be more likely in areas with stable populations under low-level chronic Ag^+^ pollution because these are the conditions in which large numbers of adults continuously occur, providing food for terrestrial predators such as birds. Conversely, severe contamination (high concentrations or pulse events near discharges) could reduce *C. riparius* populations, may decrease biodiversity [100], and rapidly change community structure and trophic organization [101].

## 5. Conclusions

This study demonstrates that chronic exposure to environmentally relevant concentrations of Ag^+^—ranging from the EU-EQS (0.01 µg/L) to higher levels (3 µg/L)—induces multigenerational and transgenerational adverse effects in *C*. *riparius*. Key findings include cumulative increases in mortality, reduced emergence and reproductive output, delayed development (particularly in females), and a decline in population growth rate over generations. Notably, transgenerational impairments persisted even after the removal of Ag^+^, especially at concentrations ≥ 0.1 µg/L. These results highlight that conventional short-term or single-generation toxicity assessments underestimate the long-term ecological risks of Ag^+^. The evidence supports the need to incorporate multigenerational testing into regulatory frameworks to better protect aquatic ecosystems from persistent pollutants such as silver and improving predictions of ecosystem recovery following pollution events.

## Data Availability

Data is contained within the article or Supplementary Materials.

## Supplementary Materials

The following supporting information can be downloaded at: https://www.mdpi.com/article/10.3390/toxics13100855/s1, Figure S1. The effect of nominal 3 µg/L Ag^+^ on Emt50 of (A) male (♂) and (B) female (♀) *Chironomus riparius* across four generations. Figure S2. The effect of nominal 3 µg/L Ag^+^ on development rate of (A) male (♂) and (B) female (♀) *Chironomus riparius* across four generations. Figure S3. The effect of nominal 3 µg/L Ag^+^ on rate of female (♀) *Chironomus riparius* across four generations. Figure S4. The effect of nominal 3 µg/L Ag^+^ on average number eggs per egg rope of *Chironomus riparius* across four generations. Figure S5. The effect of nominal 3 µg/L Ag^+^ on dry body weight of male (♂) and female (♀) *Chironomus riparius* across four generations. Figure S6. The effect of Ag^+^ (nominal concentrations 0, 0.01, 0.1, 1 and 3 µg/L) on Emt50 of (A) male (♂) and (B) female (♀) *Chironomus riparius* across seven successive generations. Figure S7. The effect of Ag^+^ (nominal concentrations 0, 0.01, 0.1, 1 and 3 µg/L) on development rate of (A) male (♂) and (B) female (♀) *Chironomus riparius* across seven successive generations. Figure S8. The effect of Ag^+^ (nominal concentrations 0, 0.01, 0.1, 1 and 3 µg/L) on rate of female (♀) *Chironomus riparius* across seven successive generations. Figure S9. The effect of Ag^+^ (nominal concentrations 0, 0.01, 0.1, 1 and 3 µg/L) on dry body weight of male (♂) and female (♀) *Chironomus riparius* across seven successive generations.
