# Supplementary material for: Multi- and Transgenerational Effects of Silver Ions (Ag+) in the ng/L Range on Life Cycle Parameters and Population Growth of the Midge Chironomus riparius (Diptera, Chironomidae)
Source: Toxics. 2025 Oct 10;13(10):855. doi: 10.3390/toxics13100855 (PMC12567948; doi:10.3390/toxics13100855)
Supplement: Supplementary file 1 [file toxics-13-00855-s001.zip › Supplementary file.pdf]

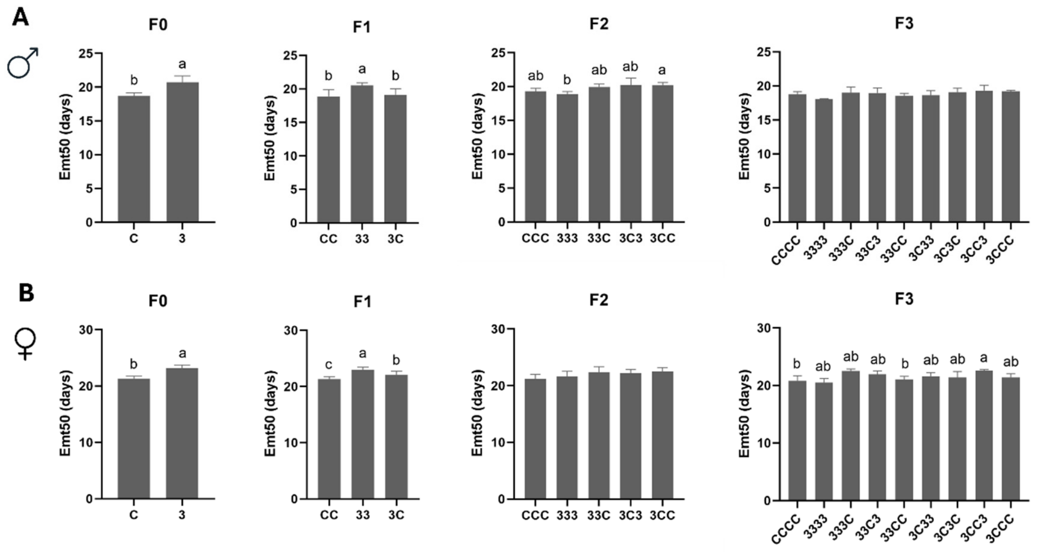

Figure S1. The effect of nominal 3 μg/L Ag^{+} on Emt50 of (A) male (♂) and (B) female (♀) *Chironomus riparius* across four generations (n = 5). Means and standard deviations. Different letters above the columns indicate significant differences ($p < 0.05$) between groups within each generation (both sexes of F0: t-test; both sexes of F1: ANOVA, LSD test; both sexes of F2–F3: Kruskal-Wallis test, Bonferroni correction for ♂ of F2 and ♀ of F3).

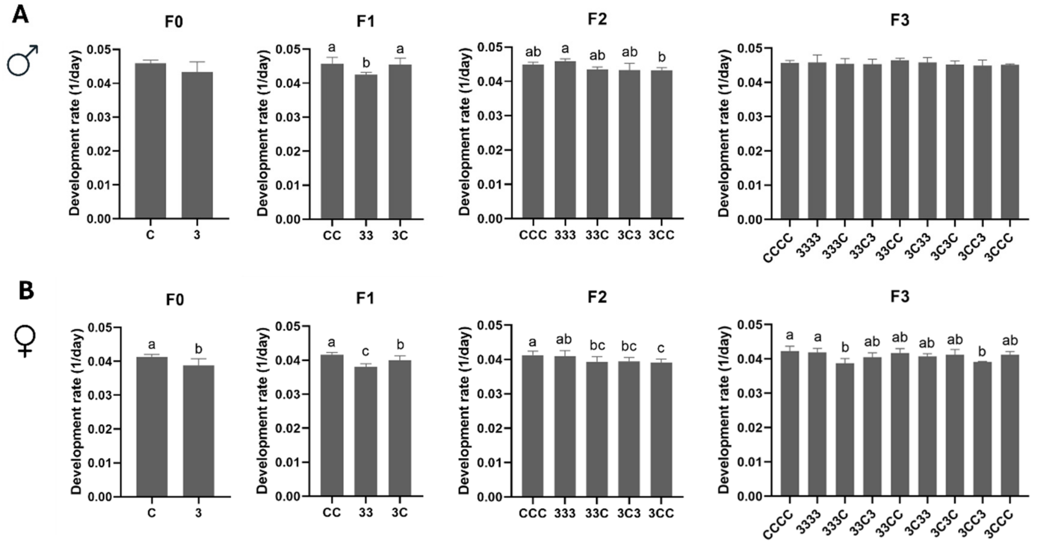

Figure S2. The effect of nominal 3 μg/L Ag^{+} on the development rate of (A) male (♂) and (B) female (♀) *Chironomus riparius* across four generations (n = 5). Means and standard deviations. Different letters above the columns indicate significant differences ($p < 0.05$) between groups within each generation (both sexes of F0: t-test; both sexes of F1: ANOVA, LSD test; F2: Kruskal-Wallis test, Bonferroni correction for ♂ and ANOVA, LSD test for ♀; F3: ANOVA, LSD test for ♂ and Kruskal-Wallis test, Bonferroni correction for ♀).

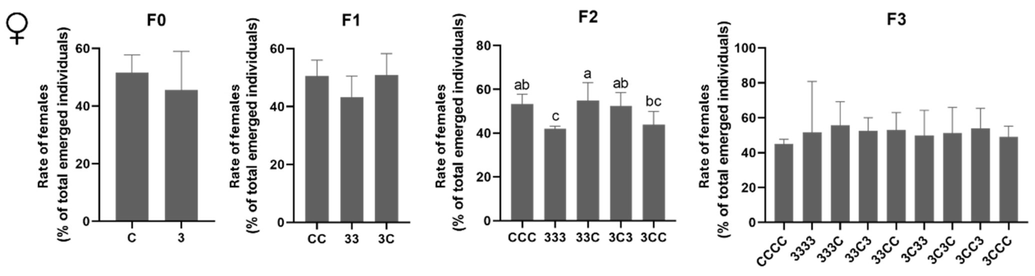

Figure S3. The effect of nominal 3 μg/L Ag^{+} on the rate of female (♀) *Chironomus riparius* across four generations (n = 5). Means and standard deviations. Different letters above the columns indicate significant differences ($p < 0.05$) between groups within each generation (F0: t-test; F1–F3: ANOVA, LSD test for F2).

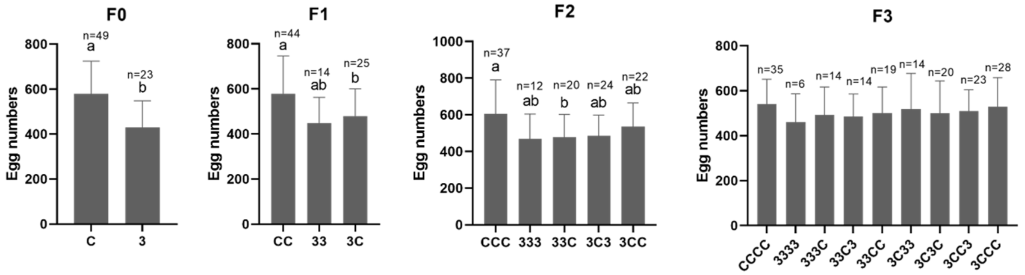

Figure S4. The effect of nominal 3 μg/L Ag^{+} on the average number of eggs per egg rope of *Chironomus riparius* across four generations. Means and standard deviations. Different letters above the columns indicate significant differences ($p < 0.05$) between groups within each generation (F0: t-test; F1–F2: ANOVA, LSD test; F3: Kruskal-Wallis test, Bonferroni correction).

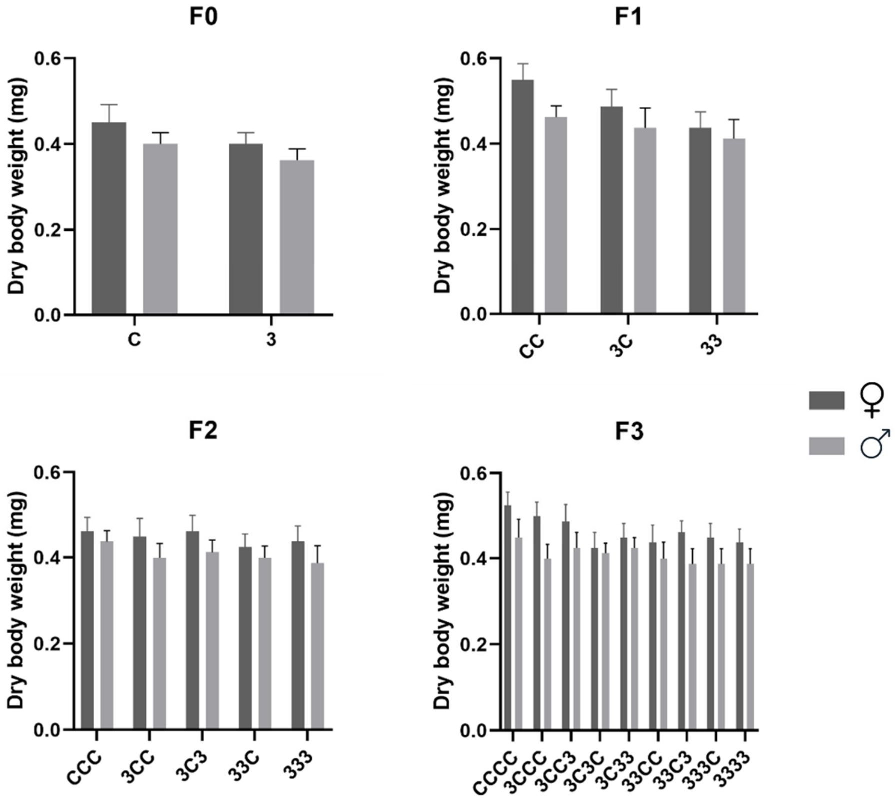

Figure S5. The effect of nominal 3 μg/L Ag^{+} on the dry body weight of male (♂) and female (♀) *Chironomus riparius* across four generations (n = 7). Means and standard deviations.

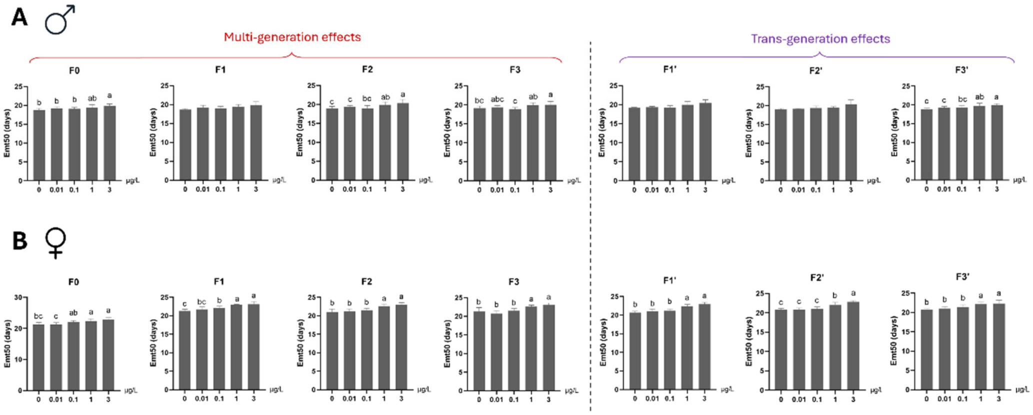

Figure S6. The effect of Ag^{+} (nominal concentrations 0, 0.01, 0.1, 1 and 3μg/L) on Emt50 of (A) male (♂) and (B) female (♀) *Chironomus riparius* across seven successive generations (n = 5), including multigenerational exposure of the first four generations (F0–F3) and transgenerational recovery of the subsequent three generations (F1′–F3′). Means and standard deviations. Different letters above the columns indicate significant differences ($p < 0.05$) between groups within each generation (For ♂: F0, F2, F3 and F3′ ANOVA, LSD test; F1′–F2′: Kruskal-Wallis test. For ♀: F0–F3 and F1′–F3′ ANOVA, LSD test).

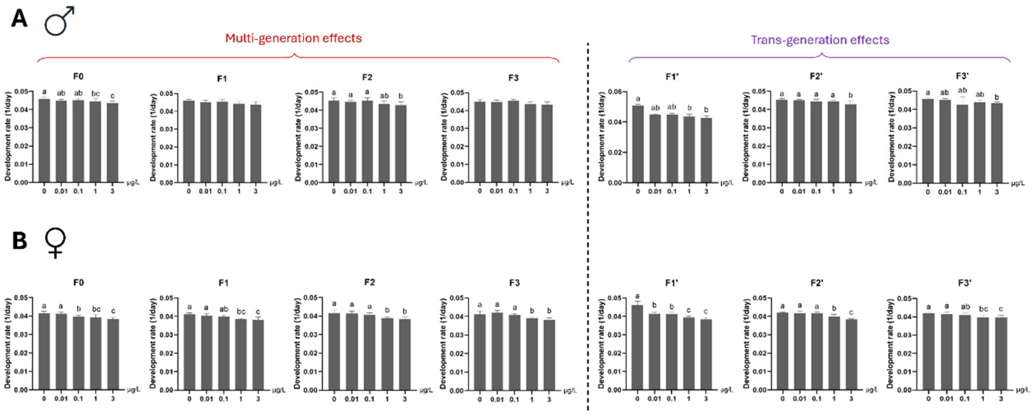

Figure S7. The effect of Ag^+ (nominal concentrations 0, 0.01, 0.1, 1 and 3μg/L) on the development rate of (A) male (♂) and (B) female (♀) *Chironomus riparius* across seven successive generations (n = 5), including multigenerational exposure of the first four generations (F0–F3) and transgenerational recovery of the subsequent three generations (F1'–F3'). Means and standard deviations. Different letters above the columns indicate significant differences ($p < 0.05$) between groups within each generation (For ♀: F0–F3 and F1'–F3' ANOVA, LSD test. For ♂: F0, F2, F3 and F2' ANOVA, LSD test; F1, F1' and F3' Kruskal-Wallis test, Bonferroni correction (the latter for F1' and F3').

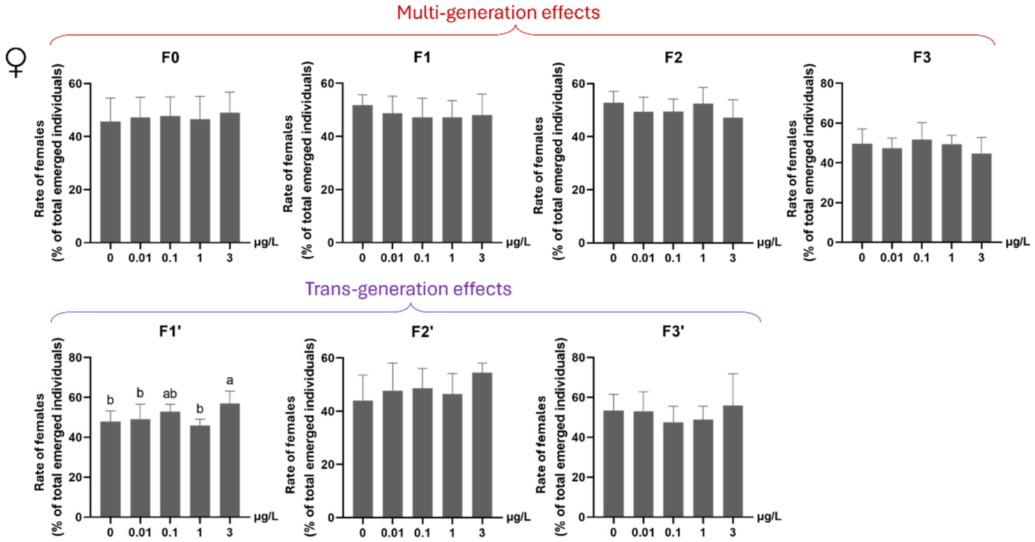

Figure S8. The effect of Ag^+ (nominal concentrations 0, 0.01, 0.1, 1 and 3μg/L) on the rate of female (♀) *Chironomus riparius* across seven successive generations (n = 5), including multigenerational exposure of the first four generations (F0–F3) and transgenerational recovery of the subsequent three generations (F1'–F3'). Means and standard deviations. Different letters above the columns indicate significant differences between groups within each generation (F0–F3 and F1'–F3': ANOVA, LSD test for F1').

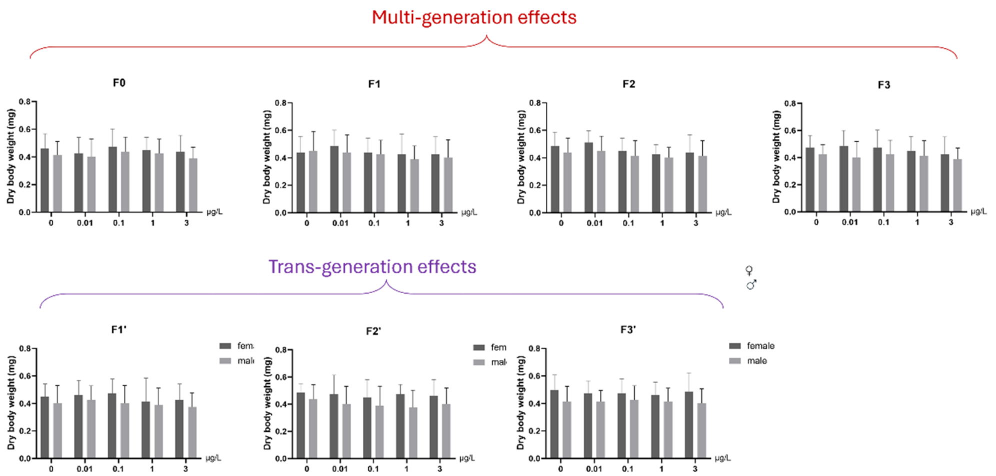

Figure S9. The effect of Ag^{+} (nominal concentrations 0, 0.01, 0.1, 1 and 3µg/L) on the dry body weight of male (♂) and female (♀) *Chironomus riparius* across seven successive generations (n = 7), including multigenerational exposure of the first four generations (F0–F3) and transgenerational recovery of the subsequent three generations (F1′–F3′). Means and standard deviations.
